# Quantitative Assessment of Asbestos Fibers in Normal and Pathological Peritoneal Tissue—A Scoping Review

**DOI:** 10.3390/life12121969

**Published:** 2022-11-24

**Authors:** Yohama Caraballo-Arias, Carlotta Zunarelli, Paola Caffaro, Francesco Roccuzzo, Mattia Roberto Nocilla, Maria Chiara Imperiale, Clara Romano, Paolo Boffetta, Francesco Saverio Violante

**Affiliations:** 1 Department of Medical and Surgical Sciences, Alma Mater Studiorum University of Bologna, 40138 Bologna, Italy; 2 School of Occupational Medicine, Alma Mater Studiorum University of Bologna, 40138 Bologna, Italy; 3 Stony Brook Cancer Center, Stony Brook University, New York, NY 11794, USA; 4 IRCCS, Azienda Ospedaliero-Universitaria di Bologna, 40138 Bologna, Italy

**Keywords:** asbestos fibers, peritoneal mesothelioma, electron microscopy, occupational diseases, chrysotile, amphiboles, scoping review

## Abstract

Peritoneal tissue is the second most affected site by malignant mesothelioma linked to asbestos exposure. This scoping review aims to summarize the findings of the studies in which asbestos fibers in the peritoneum were quantified by electron microscopy, occasionally associated with spectroscopy, both in neoplastic and non-neoplastic tissue. The 9 studies selected comprised 62 cases, out of whom 100 samples were analyzed. Asbestos fibers were detected in 58 samples (58%). In addition, 28 cases had diagnosis of peritoneal mesothelioma. For 32 cases, a lung tumor sample was available: 28/32 samples analyzed presented asbestos fibers; 18/32 reported amphiboles with a range from not detected to 14.2 million fibers per gram of dry tissue (mfgdt); 18/32 reported chrysotile, with a range of 0 to 90 mfgdt. The studies were heterogeneous for type of samples, analytical technology, and circumstances of exposure to asbestos. To evaluate asbestos fibers in the peritoneum and to better understand the association between asbestos exposure and malignant peritoneal mesothelioma, it is desirable that the search for asbestos fibers becomes a routine process every time peritoneal tissue is accessible.

## 1. Introduction

Asbestos is a commercial definition for a group of crystalline mineral silicates that occur naturally in various forms [1]. There are various morphological types that differ in their mechanical, chemical, and toxicological properties [2,3]. The fibers that characterize these minerals are longitudinal structures with a ratio of length to width of at least 3:1 [4] and, based on their chemistry and morphology, are divided into two groups: amphiboles (Crocidolite, Amosite, Tremolite, Actinolite, and Anthophyllite) and the serpentine group (chrysotile), which represents 90–95% of all the asbestos worldwide. Because of its structure, the serpentine class is more easily cleared by mucociliary action and broken down, while the amphibole class is more resistant to clearance from the body, with a longer residence time.

Asbestos fibers, especially amphiboles, have a long persistence in tissues and their presence has been studied for almost 50 years in humans, especially with electron microscopy (scanning—SEM or Transmission—TEM) [5].

From 1950 to 1985, it was extensively used in construction and shipbuilding for insulation and fire protection and for friction materials and filters. Any building constructed during this period might contain some type of asbestos-containing material, which is possibly the most important cause of inadvertent exposure. A drastic reduction in using asbestos in European countries has taken place in the last decade.

Of the three types most commonly used, and now found in construction work (especially chrysotile), all cause severe health effects, although amosite and crocidolite fibers are recognized as being more dangerous to health than chrysotile [2,6].

Another source of asbestos in the air is from anthropogenic deposits, such as mining operations, crushing, screening, and milling of the ore; manufacturing asbestos products; use of asbestos-containing materials: transport and disposal of wastes containing asbestos; demolition of buildings constructed with asbestos-containing products, such as insulation, fireproofing, ceiling and floor tiles, roof shingles, drywall, and cement [7].

Health effects of asbestos exposure are well known, especially the damage to lung and pleural tissues. Likewise, malignant mesothelioma is a rare neoplasm arising in the serosal lining of body cavities, the annual incidence of malignant peritoneal mesothelioma (MPeM) has been reported to be 0.2 to 3 cases per 1,000,000 people per year, globally. Although the pleura is the most common primary site, ~10% of mesotheliomas arise in the peritoneum [8,9]; pericardial and para-testicular mesothelioma each account for <1% of cases [9].

The incidence of peritoneal mesothelioma in the United States, Western Europe, and Australia has remained relatively stable for 40 years, at ~0.04–0.11 new diagnoses per 100,000 person-years among women, and 0.07–0.16 per 100,000 person-years among men [10,11].

Mesotheliomas arising at different sites have distinct morphologic, molecular, and clinical profiles. Compared to pleural mesothelioma, peritoneal tumors occur more often in younger patients and women, are less often linked to asbestos exposure, and more often show epithelioid histotype [8,12,13,14]. However, given its relative rarity, peritoneal mesothelioma has not been as well characterized as pleural mesothelioma.

Malignant mesothelioma is linked to exposure to asbestos in a large proportion of cases, most of which concern pleural tissue (73–85%), with the peritoneum being the second most affected site (7–18% of total cases) [7,15].

Malignant peritoneal mesothelioma develops predominantly as expansive more than infiltrative neoplasm. Symptoms are related to the extent of tumor spread within the abdominal cavity. They are not specific, occurring in over 30–50% of patients and including abdominal pain and distention (for example, because of ascetic fluid or intestinal obstruction). Other symptoms include weight loss, abdominal mass, anorexia, and a new onset abdominal wall hernia. Because of an unspecific clinical pattern, its diagnosis is often incidental, either found on cross-sectional imaging or during abdominal laparoscopy or laparotomy [16].

As predictable, the non-specific character of the symptoms can lead to a diagnosis of malignant peritoneal mesothelioma at a later stage [16].

The evaluation (both quantitative and qualitative) of fibers in the target tissue is used to establish the intensity of the exposure; this can be performed via electron microscopy occasionally associated with spectroscopy [17,18,19].

Studies in which asbestos fibers have been determined in peritoneal tissues are scarce, as most of the scientific literature focuses on studies of asbestos fibers in lung tissue.

The aim of this scoping review is to summarize the studies in which asbestos fibers were quantified through various analytical techniques in the peritoneum, both in neoplastic and non-neoplastic tissue.

## 2. Materials and Methods

We performed a detailed review of published literature by interrogating three main scientific databases. The search strategy was based on a PubMed search filter conceived to detect papers on which the asbestos exposure was determined by detecting fibers on tissues by electron microscopy.

The inclusion criteria for this review were:

- Articles written in any language, regardless of the publication date.
- Articles reporting a quantification of asbestos fibers in human peritoneal tissue including the greater omentum and mesentery (whether normal or pathological) by means of electronic microscopy in subjects with defined or undefined asbestos exposure.
- The exclusion criteria were:
- Articles not reporting a quantitative measure of the number of asbestos fibers found in human peritoneal tissue or reporting a measure by techniques other than electron microscopy.
- Studies in non-human subjects or in tissues other than the peritoneum.

Initially, we searched through three databases: PubMed, Scopus, and Embase, and we used the same terms “asbestos * AND electron * AND peritone *” on 4 February 2022. We identified 159 articles from PubMed, 151 from Scopus, and 147 from Embase. After eliminating duplicates, 233 articles remained and were independently evaluated by two of the authors (CZ and YC); 69 articles were discarded after reading the title and the abstract, and 152 after checking the full text. The references of each relevant article were manually searched, yielding no more papers; finally, we identified 9 studies that fitted our inclusion criteria [20,21,22,23,24,25,26,27,28]. A total of 8 of the studies were published between 1989 and 2002, and only 1 after 2002.

A further article by Suzuki et al. [29] fitted the inclusion criteria; however, the authors analyzed the same cases reported in the study by Kohyama and Suzuki [22], published in the same year (1991).

## 3. Results

Table 1 reports a description of the patients and conditions included in the studies. Table 2 describes the detection limits of asbestos fibers in the peritoneal tissue (expressed as 10^6^ fibers per gram of dry tissue) and the analytical technology used. When the detection limits were not specified in the study, we listed the lowest concentration of asbestos fibers reported. Table 3 reports the number of asbestos fibers found in each sample.

In the study by Dodson et al. [25], case number 19 was considered an outlier because asbestos fibers were not present in any samples analyzed, not even in the lung.

The 9 studies included in this review comprised 62 cases, in whom 100 samples were analyzed. The studies were performed on autopsy samples or biopsies. In addition, 5 studies did not specify if the samples were obtained from autopsies or living patients [22,24,26,27,28].

The articles included in the review did not have enough data to make a comparison between cases of mesothelioma, non-mesothelioma, and control cases. Moreover, many studies did not specify the origin of the analyzed anatomical samples. Six of the studies analyzed peritoneal mesothelioma [20,21,22,24,27,28], without specifying the section of the organ (anatomical site); the omentum (normal and pathological) was analyzed in 3 studies [23,25,26] and the mesentery in 2 studies [25,26].

Asbestos fibers were detected in 58 samples (58%) and were below the detection limit in 42 samples (42%). Most of the studies reported both chrysotile and amphiboles fibers [22,23,24,25,26,27], 2 studies did not report any fibers at all [20,28], and only 1 study reported only chrysotile asbestos [21].

The studies with the highest number of cases and samples were published by Dodson et al. (19 cases) [25], followed by the same authors in a successive paper (15 cases) [25,26].

Of the 62 cases, 28 were diagnosed with peritoneal mesothelioma (45%) and 16 with pleural mesothelioma (26%). Other diagnoses were unrelated to asbestos exposure in 18 cases (29%) [23,26]. Table 4 summarizes the range and type of fibers found in peritoneal tissue analyzed in peritoneal mesothelioma cases, including the greater omentum and mesentery [23,25]. Saitoh et al. [23] reported the range from observations on 3 samples prepared per tissue; therefore, a precise quantification of samples was not ascertainable, and the highest value was reported.

A total of 32 samples of tumoral tissue were taken from the 28 mesothelioma cases. Asbestos fibers were detected in 28/32 samples of tumoral tissue (87%), while in 4/32 samples, they were below the detection limit (13%). The number of samples with amphiboles was the same as the number of samples with chrysotile. Amphibole fibers were present in 56% of 32 samples of peritoneal mesothelioma. The lowest value of amphibole fibers was not always quantified and, generally, it was defined as “not detected”; therefore, the amphiboles ranged from 0.000447 mfgdt to 14.2 mfgdt. Chrysotile was detected in 56% of 32 samples, and by following the same directions as described for amphiboles, the range was from the lowest positive value of 0.33 mfgdt to 90 mfgdt. Amphiboles were found in 4 out of 8 studies, in 18 samples out of 32, and chrysotile was found in 4 out of 8 studies, in 18 samples out of 32. In 2 studies, asbestos was not found [20,28], and in 3 studies [20,23,28], the type of fibers was not differentiated. Horie et al. [20] reported a case of peritoneal malignant mesothelioma in a radiation technologist. Peritoneal mesothelioma can be induced by therapeutic radiation [30,31,32]. The patient had no history of exposure to asbestos. An autopsy was performed and a sample of retroperitoneal tumoral tissue was taken. During the autopsy, it was pointed out that the irregular thickening of the intestinal serosa was prominent and was imputed as the cause of death (ileus). Hence, we reported in Table 1 retroperitoneal mesothelioma as a type of diagnosis. The authors did not find asbestos fibers in peritoneal tissue, but they found asbestos in the lung. The patient was exposed to a low dose of occupational radiation, for which the authors agreed that the etiology of the peritoneal mesothelioma remained unknown.

Morinaga et al. [21] analyzed in a preliminary report 23 mesotheliomas, 5 rejected cases, and 17 controls out of 49 autopsies. The group of 23 mesotheliomas comprised 21 pleural, 1 pericardial, and 1 peritoneal case. The samples were examined for asbestos semiquantitative fiber content with a TEM and EDS (Table 2). In 19 samples, out of the 23 mesotheliomas, asbestos fibers were found in the pleura, pericardium, and peritoneum. In the only peritoneal mesothelioma case, a few fibers were found in the entire specimen, and it was not possible to establish any occupational exposure to asbestos. As reported in Moringa et al., there was one case of peritoneal mesothelioma analyzed and only chrysotile fibers were found.

Kohyama and Suzuki [22] evaluated 33 samples from 13 individuals. All were males with a diagnosis of asbestosis, lung cancer, and/or mesothelioma (either pleural or peritoneal). Six samples were directly taken from peritoneal mesothelioma. All cases had a specified asbestos exposure duration (25 to 53 years). The detection limit of asbestos fibers in peritoneal mesothelioma was 0.17 mfgdt. All the peritoneal tumors had chrysotile, ranging from 12.6 to 89.6 mfgdt; three of the samples had amosite fibers, ranging from 0.52 to 14.2 mfgdt. No other type of fibers was detected. One finding was that the burden of chrysotile fibers was similar in both lung parenchyma and mesotheliomas, unlike other types of fibers.

Saitoh et al. [23] reported 2 patients diagnosed as malignant peritoneal mesothelioma [15] and 3 patients from the general population (control cases) who died from other causes. The cases were 2 males and 3 females. Three samples per tissue were prepared. The range reported in Table 2 of that article showed data from 3 observations. Some samples were not analyzed. Case 1 of their case study had a diagnosis of peritoneal mesothelioma and a probable occupational exposure to asbestos, and case 3 (control case) with another diagnosis had a possible occupational exposure to asbestos. In case number 1, asbestos fibers were found in tumor or non-tumor tissues and their concentrations in tumoral tissue were comparable to the lung tissue levels. Case 2 had a diagnosis of peritoneal mesothelioma and an unlikely occupational exposure to asbestos; a few fibers were also detected in tumoral tissues of the greater omentum. In case 2, many silica particles were included (defined fibrous substances of needle-type); asbestos fibers were similar in shape to the fibers of the remaining cases but were present at lower concentration.

Heller et al. [24] analyzed 7 peritoneal malignant mesotheliomas, confirmed by immunohistochemical evaluation; prior to surgery, the cases were identified as ovarian carcinoma. All cases were females and there was no known asbestos exposure. Asbestos fibers were detected in 6 of 7 cases. In Table 2 of their study, the patient’s age and asbestos fibers type were reported. Specifically, in 2 cases, crocidolite was found; in 2, chrysotile; in 1, chrysotile and amosite; in 1, chrysotile and tremolite. The authors concluded that the etiology without a known history of exposure to asbestos was unclear. They postulated alternative routes of exposure to asbestos, such as the transvaginal route, which allows for a lower intensity of exposure compared to the intensity usually needed in the lung, to induce pathological changes in the peritoneal cavity (peritoneum and ovary).

Dodson et al. [25] analyzed tissue from 20 individuals with mesotheliomas: 17 had diagnosis of pleural mesothelioma and 3 had diagnosis of peritoneal mesothelioma. All cases were male and most had an occupation with an expected exposure to asbestos. Autopsies were performed and samples from the lung, omentum, and mesentery were taken for each patient. The authors reported that case number 19 had no fibers found as based on the detectable limits within the study; as it had no values reported in any tissue analyzed, it was defined as an exception in contrast to the other cases. For this reason, we did not include it in the overall analysis. In the analytical transmission electron microscopy (ATEM), asbestos bodies were found in the mesentery in five individuals (two out of the five were peritoneal mesotheliomas). In the ATEM scan, asbestos bodies were found in the mesentery in five individuals (two out of the five were peritoneal mesotheliomas). Only two cases were found to have asbestos bodies in the omentum. As expected, the highest number of asbestos bodies in the mesentery and in the omentum was found in the patients with the highest number of asbestos bodies in the lung tissue. A counting of asbestos fibers was reported in Table 2 of their paper. Chrysotile and amphiboles reached the omentum in several cases, which showed that asbestos fibers are translocated and could be potentially important in the pathogenesis of peritoneal mesothelioma.

Dodson et al. [26] took samples from 15 individuals (9 males and 6 females, 10 to 59-year-old) in East Texas, none of whom had occupational exposure to asbestos. The samples had to be collected so that another sample, adjacent to the one subject of study, was free of histologically relevant changes (which may infer a pathological process going on) and so that the ferruginous body burden in the lungs was compatible with the general population. A total of 15 samples were taken from the omentum and from the mesentery. All the measurements considered wet tissue. No ferruginous bodies were observed in the omentum nor in the mesentery and no uncoated asbestos fibers were found in the mesentery; in 2 different samples of the omentum, 146 and 145 million fibers per gram of wet tissue, thus converted to 174 and 176 mfgdt, were found, with the detection limit being, respectively, 174 and 176 mfgdt.

Suzuki and Yuen [27] evaluated 168 cases of malignant mesothelioma (164 males and 4 females); 12 of the overall mesotheliomas involved the peritoneum. Six samples were directly collected in the cancerous peritoneum and one was collected in the transitional zone between tumor and plaque. All the cases analyzed had occupational exposure, all of them being insulation workers. It is not known for how long the workers had been exposed to asbestos. The samples were 0.1 to 0.2 g in their dry state. All the samples presented chrysotile fibers, ranging from 90 to 12.6 mfgdt; 3 of the samples had amosite, ranging from 0.52 to 14 mfgdt. No other fibers were detected; the lowest detection limit of asbestos fibers was 0.17 mfgdt and the highest was 1.42 mfgdt. The highest total value detected was 104 mfgdt, while the lowest was 14.4 mfgdt. One conclusion was that the number of chrysotile fibers was 30 times greater than the number of amphibole fibers in mesothelial tissue and, therefore, a likely implication that chrysotile was more prone to cause mesothelioma was inferred.

Hung et al. [28] analyzed 88 peritoneal mesotheliomas in 39 men and 49 females. In this series of peritoneal mesotheliomas, the authors identified ALK-positive mesotheliomas by immunohistochemistry and confirmed ALK rearrangement by FISH (fluorescence in situ hybridization). The authors found unique ALK rearrangements in 3 peritoneal mesotheliomas. These samples were the only ones in which the asbestos fibers were quantified by combined scanning electron microscopy and X-ray spectroscopy. No asbestos fibers were found in these samples.

## 4. Discussion

One hundred samples from the mesentery, omentum, and mesothelioma were taken out of sixty-two patients. Asbestos fibers were detected in fifty-eight samples (58%), mainly reporting both chrysotile and amphibole fibers, and in forty-two samples (42%), fibers were not detected.

Twenty-eight out of sixty-two cases (45%) had diagnosis of peritoneal mesothelioma (45%). Focusing on them, thirty-two samples of tumoral tissue were analyzed: 28/32 reported asbestos fibers (87%) and 4/32 were negative for asbestos fibers (13%). In the positive samples, amphiboles were detected in 18/32 with a range from not detected to 14.2 mfgdt; chrysotile was detected in the same number of samples with 18 positives out of 32, ranging from 0 to 90 mfgdt. Despite numerically overlapping, not always in the same tissue analyzed, both amphiboles and chrysotile were present. The findings suggest that asbestos fibers quantification may be useful in the identification of asbestos-related peritoneal malignant mesothelioma.

Asbestos is a carcinogen implicated in the pathogenesis of peritoneal mesothelioma. Although the association with asbestos exposure is weaker than in pleural mesothelioma, asbestos is one of the risk factors for this disease [16].

Malignant peritoneal mesothelioma is a rare malignant disease that develops from serosal surfaces of the peritoneal cavity. Early diagnosis is made based on cross-sectional imaging and a definitive diagnosis can be established on a combination of morphologic and immunohistochemical features derived from tissue biopsy [33].

In fact, biopsy is essential for establishing diagnosis (performed either radiographically or surgically) and laparoscopy represents a preferable diagnostic approach, considering its lower invasiveness and clear intraoperative assessment [34].

There still is much debate about how asbestos fibers might enter the abdomen and extensive research has been directed toward studying the role of asbestos fibers on cancerogenesis [35].

To develop and extend knowledge on this topic, a significant contribution can come from the correlation between the intensity of the exposure and the quantitative and qualitative evaluation of asbestos fibers in the peritoneum.

As for asbestos fiber determination in the serosa, there could be a “true negative” because fibers are absent in the peritoneal tissue sampled, but may be present in the adjacent zone, especially if the peritoneal tissue analyzed is based on a small sample. The analytical results could also be a “false negative” for various reasons, including samples preparation or microscopic processing [5].

The studies included in our review are heterogeneous: as for the samples considered (autopsy samples and biopsies), the analytical technology used, and the exposure to asbestos. Taking into consideration that the articles reviewed are all case series, the level of evidence of this review remains at this stage.

The electron microscopy technology has relevant implications in the quantification of asbestos fibers. Most of the studies were published over 20 years ago; thanks to technological advancement, asbestos fibers may be recognized better today than when the original analyses were run, even using automated techniques.

To better evaluate the presence of asbestos fibers in the peritoneum and, thus, to better quantify the association between asbestos exposure and malignant peritoneal mesothelioma, it is desirable that the search for asbestos fibers becomes a routine process during interventions in which peritoneal tissues are more easily accessible, such as biopsy and surgery. To obtain the best results, using the most advanced technologies and training laboratory personnel can be very helpful.

However, it is appropriate to consider a multifactorial etiopathogenesis of cancer. Specifically, genomic and immunophenotypic features play a key role in mesothelioma development. Distinct mesothelioma biology and genomic characteristics could modify the prognosis and overall survival [36].

Thus, we suggest quantifying asbestos fibers every time peritoneal tissue is accessible and to correlate the presence of the mineral with occupational and environmental asbestos exposure. All this information will contribute to broaden the knowledge of a neoplasm that is still poorly characterized and to increase the understanding of asbestos-related carcinogenicity [37].

## Figures and Tables

**Table 1:** Description of the patients and diagnoses in the included studies.

Reference	N° of Cases	Type of Diagnosis
Horie et al., 1989 [20]	1 autopsy	1 retroperitoneal mesothelioma
Morinaga et al., 1989 [21]	1 autopsy	1 peritoneal mesothelioma
Kohyama and Suzuki., 1991 [22]	5 *	5 peritoneal mesothelioma
Saitoh et al., 1993 [23]	5 autopsies	2 peritoneal mesothelioma 3 other causes
Heller et al., 1999 [24]	7 *	7 peritoneal mesothelioma
Dodson et al., 2000 [25]	19 autopsies	16 pleural mesotheliomas 3 peritoneal mesotheliomas
Dodson et al., 2001 [26]	15 *	15 other causes <<a companion tissue sample adjacent to that taken for fiber burden analysis was determined to be free (by light microscopy of tissue sections) of pathological changes, which might suggest asbestos-induced disease>>
Suzuki and Yuen, 2002 [27]	6 *	6 peritoneal mesothelioma
Hung et al., 2017 [28]	3 *	3 peritoneal mesotheliomas

* Not reported if autopsy or biopsy.

**Table 2:** Detection limit of asbestos fibers in the peritoneal mesothelioma, expressed in number of fibers × 10^6^.

Reference	Detection Limit for Amphiboles (10^6^/Gram of Dry Tissue) *	Detection Limit for Chrysotile (10^6^/Gram of Dry Tissue) *	Technology Used
Horie et al. [20]	Not available	Not available	Electron microscope
Morinaga et al. [21]	Not available	Not available	TEM and EDS
Kohyama and Suzuki [22]	0.17	0.17	TEM with fluorescence screen with a light microscope and EDS
Saitoh et al. [23]	12	12	SEM and EDX
Heller et al. [24]	0.33	0.11	TEM, EDS, and SAED
Dodson et al. [25]	0	0	ATEM and EDX
Dodson et al. [26]	0	0	ATEM and EDX
Suzuki & Yuen [27]	0.17	0.17	Electron microscope with EDX
Hung et al. [28]	Not available	Not available	SEM and EDS

* When the DL was not specified, the lowest value observed is reported. TEM = Transmission electron microscope; EDS = energy-dispersive X-ray spectroscopy; EDX = Energy-Dispersive X-ray Analysis; SAED = Selected-area electron diffraction; ATEM = Analytical Transmission electron microscope; SEM = Scanning electron microscope.

**Table 3:** Asbestos fibers found in analyzed samples (for additional details, the readers are referred to the synthesis of the studies in the text).

Reference	N° of Subjects/n° of Samples°	Type of Tissue Analyzed	Asbestos Exposure	Type of Asbestos Found (N° of Samples with/without Fibers)	N° of Asbestos Fibers Per Gram of Dry Tissue *		
					Median * ^2^	Range	IQ Range
Horie et al. [20]	1/1	(Retro) Peritoneal mesothelioma	Unexposed	Total (0/1)		0	
Morinaga et al. [21]	1/1	Peritoneal mesothelioma	Unknown	Chrysotile (1/0)		(+) * ^3^	
Kohyama and Suzuki [22]	5/6	Peritoneal mesothelioma	Occupational	Amphiboles (4/2)		Not detected–14.2 × 10^6^	
				Chrysotile (6/0)		12.6 × 10^6^–89.6 × 10^6^	
Saitoh et al. [23] * ^4^	5/7	Greater omentum (tumoral)	Occupational	Total (1/0)		Only one value (36 × 10^6^)	
			Unlikely or unknown	Total (1/0)		Only one value (12 × 10^6^)	
		Greater omentum (normal)	Occupational	Total (2/0)		12 × 10^6^–72 × 10^6^	
			Unlikely or unknown	Total (1/2)		Not detected–12 × 10^6^	
Heller et al. [24]	7/7	Peritoneal mesothelioma	Unexposed	Amphiboles (4/3)		0.33 × 10^6^–3.78 × 10^6^	
				Chrysotile (4/3)		0.33 × 10^6^–19.63 × 10^6^	
Dodson et al. [25]	19/19	Omentum	Occupational	Amphiboles (14/5)	224	0–6553	0–1644
				Chrysotile (3/16)		0–1029	
	19/19	Mesentery	Occupational	Amphiboles (15/4)	447	0–5445	173–2971
				Chrysotile (5/14)		0–743	
Dodson et al. [26]	15/15	Omentum	Unexposed	Amphiboles (1/14)	0	0–176	0–0
				Chrysotile (1/14)	0	0–174	0–0
	15/15	Mesentery	Unexposed	Amphiboles (0/15)		0	
				Chrysotile (0/15)		0	
Suzuki and Yuen [27]	5/6	Peritoneal mesothelioma	Occupational	Amphiboles (4/2)		Not detected–14 × 10^6^	
				Chrysotile (6/0)		12.6 × 10^6^–90 × 10^6^	
	1/1	Tumor/plaque (mixed)	Occupational	Amphiboles (0/1)		Not detected	
				Chrysotile (1/0)		Only one value (17 × 10^6^)	
Hung et al. [28]	3/3	Peritoneal mesothelioma	Not reported	Total (0/3)		Not detected	

* When original data were reported for wet tissue, the results were multiplied by 10 to convert them to dry tissue. * ^2^ Median and inter quartile (IQ) range were calculated only for cells containing 10 values or more. * ^3^ In the paper, it was reported that “the amounts of asbestos fibres observed were graded as (+) a few fibres found in the entire specimen”. * ^4^ Saitoh et al. reported the range from observations on three samples prepared per tissue. We considered the highest value reported and we did not count the samples not analyzed.

**Table 4:** Range and type of fibers found in peritoneal tissues analyzed in peritoneal mesothelioma cases.

References	Type of Tissue	N° of Samples with/without Fibers	Type of Asbestos Found	Range of Asbestos Fibers/Gram of Dry Tissue
Horie et al. [20]	Retroperitoneal mesothelioma	0/1	Total	Not detected
Morinaga et al. [21]	Peritoneal mesothelioma	1/0	Chrysotile	Only one value (+) *
Kohyama and Suzuki [22]	Peritoneal mesothelioma	4/2	Amphiboles	Not detected–14.2 × 10^6^
		6/0	Chrysotile	12.6 × 10^6^–89.6 × 10^6^
Saitoh et al. [23]	Peritoneal mesothelioma	2/0	Total	12 × 10^6^–36 × 10^6^
Heller et al. [24]	Peritoneal mesothelioma	4/3	Amphiboles	0.33 × 10^6^–3.78 × 10^6^
		4/3	Chrysotile	0.33 × 10^6^–19.63 × 10^6^
Dodson et al. [25]	Peritoneal mesothelioma (omentum)	3/0	Amphiboles	852–2555
		0/3	Chrysotile	0
	Peritoneal mesothelioma (mesentery)	3/0	Amphiboles	447–3009
		0/3	Chrysotile	0
Suzuki and Yuen [27]	Peritoneal mesothelioma	4/2	Amphiboles	Not detected–14 × 10^6^
		6/0	Chrysotile	12.6 × 10^6^–90 × 10^6^
	Tumor/plaque (mixed)	0/1	Amphiboles	Not detected
		1/0	Chrysotile	Only one value (17 × 10^6^)
Hung et al. [28]	Peritoneal mesothelioma	0/3	Total	Not detected

* In the paper, it was reported that “the amounts of asbestos fibres observed were graded as (+) a few fibres found in the entire specimen”.
